# Caregiver Socio-Economic Factors and Perceived Effectiveness of Care Delivery in Relation to US Adolescent Vision Care: A Retrospective Analysis from a National Database

**DOI:** 10.3390/pediatric17050088

**Published:** 2025-08-31

**Authors:** Erik Miron, Nada Eldawy, Ayden Dunn, Austin Lent, Lea Sacca

**Affiliations:** Charles E. Schmidt College of Medicine, Florida Atlantic University, Boca Raton, FL 33487, USA; emiron2024@health.fau.edu (E.M.); neldawy2022@health.fau.edu (N.E.); adunn2023@health.fau.edu (A.D.); alent2018@health.fau.edu (A.L.)

**Keywords:** adolescent vision screening, annual exam, patient-provider communication, insurance coverage, United States

## Abstract

**Objective:** The objective of this retrospective cross-sectional study is to explore how caregiver social determinants of health, appraisal of healthcare provider effectiveness, and insurance coverage influence caregiver ability to have their adolescent child access vision care, including completion of annual vision screening, visiting an ophthalmologist or optometrist, and completion of recommended additional screenings. **Study Design:** We used National Survey of Children’s Health (NSCH) data for 12–17-year-old adolescents for the years 2022 and 2023 (n = 37,425). Summary statistics for the selected sample were generated and binary logistic regressions were conducted. Outcome variables were the type of vision screening that occurred or not. Covariates were socioeconomic and demographic data of the adolescent’s primary caregiver. Independent variables were insurance coverage and healthcare provider’s skill and effectiveness. **Results:** Significant associations were reported between visiting an ophthalmologist or optometrist and each of spending enough time with patients; listening carefully to patients; and making patients feel like care is a partnership. Additionally, significant associations were reported between insurance coverage and both successful completion of vision screening and visiting an eye doctor. **Conclusions:** This study underscores the substantial impact of effectiveness of eye doctors in delivering annual vision exams and insurance adequacy on adolescent vision care engagement. Our results will inform the development of future evidence-based educational interventions to raise awareness on the importance of annual vision screenings in US adolescents and emphasize the need for screening mandates to advocate for this important public health issue.

## 1. Introduction

Visual impairments were estimated to affect approximately 1.2 million adolescents (<18 years of age) in the United States with a persistently upward trend in diagnoses between the years 2010 to 2019 [1]. Other estimates using the National Health and Nutrition Examination Survey (NHANES) show that up to 12.3% of U.S. adolescents aged 12–18 had a distance visual impairment, which was defined as, at best, 20/40 in their better-seeing eye [2]. The prevalence of correctable vision loss has also been found to have increased within pediatric, school-aged populations during COVID-19 home confinement period beginning in 2019 [3]. Notably, one study found the burden of myopia significantly increased in 2020 compared to cohorts from the five years prior. This concerning trend, likely driven by a combination of insufficient time spent on outdoor activities and increased screen time, contributes to the growing burden of visual impairment in today’s adolescent populations compared to earlier estimates [4].

The most common visual impairments reported in this age group are uncorrected refractive errors, including myopia, hyperopia, and astigmatism, which can occur at any age but is more frequently developed in younger childhood and can be a lasting contributor to amblyopia [5,6]. Vision screening is often conducted in pediatric settings, though these protocols vary based on state and federal mandates for vision screening and are more often carried out in pre-school and school-aged populations [5]. The components of vision screening can include traditional subjective visual acuity tests; ocular alignment and stereoacuity testing; color vision testing with pseudoisochromatic plates; and, less frequently, instrument-based screening using technology such as photoscreening or autorefraction devices [5]. A study plotting five-year trends in U.S. pediatric vision screening and access found that the prevalence of vision screening has been steadily and significantly declining since 2016, and, despite a modest increase in blindness and trouble seeing when wearing glasses in the same period, there was a significant increase in unmet vision care after the onset of the COVID-19 pandemic [7]. Other studies cite testing rates dropping significantly after early elementary years, a matter compounded by the fact that, without a federal standard, only 19 U.S. states require vision screening during high school years, contributing to geographic differences in screening access [8,9].

The most recent Bright Futures/American Academy of Pediatrics Recommendations for Preventative Pediatric Healthcare recommends vision screening biennially from ages 6–15. For adolescents aged 16 to 21 years, routine vision screening is no longer recommended unless there are specific concerns or risk factors, after which a risk assessment would be performed and a comprehensive eye examination conducted only if issues are identified in the assessment [10,11]. However, based on the 2021 National Health Interview Survey (NHIS), adolescents from 16–17 years old had higher odds of vision difficulty when compared to children less than five years despite having less frequent screening requirements [12]. Data from the 2023 National Survey of Children’s Health also showed higher percentages of blindness (significantly impaired vision) or problems seeing, even when wearing glasses, in the 12–17-year-old group compared to groups 0–5 years and 6–11 years. Despite this, 39.9% of the 12–17-year-olds surveyed nationally had not received a vision screening in the past two years [13].

There is strong evidence that leaving such impairments undiagnosed or untreated can have lasting social and health consequences. Adolescents experiencing severe visual impairments were significantly less likely to meet national guidelines for physical activity compared to their peers without visual impairments, placing them at increased risk of obesity and poorer general health outcomes [14,15,16,17]. A study interviewing youth with bilateral visual impairments and their parents identified concerns over academic performance and social involvement in activities or sports [18]. Research conducted in various European countries examining quality of life in adolescents with visual impairments echoed these concerns, finding that lack of functional vision was associated with decreased quality of life, social withdrawal, and lower perceptions of acceptance among their peers [19,20,21,22,23]. In the U.S., vision concerns were found to be more common among female, low-income, and uninsured adolescents, and was significantly associated with recent poor mental well-being [24]. A scoping review also found a higher prevalence of depression and anxiety in adolescents with visual impairments compared to normally sighted peers, underscoring the unseen consequences of ocular morbidity on this age group [25].

Beyond legislation regarding screening guidelines in adolescence, sociodemographic barriers have been examined with respect to vision testing and annual screenings, several of which are related to parental factors and affordability [8,12]. Insurance and poverty status both directly influenced the screening rates in children and adolescents. Moreover, adolescents without a primary care provider, living under the poverty line, and those who were either Medicaid-insured or uninsured were significantly less likely to receive vision testing and more likely to have undiagnosed and untreated visual impairments [8,26,27,28,29]. Several studies have also examined racial and ethnic differences in access to eye care and risk of visual impairment, finding that Black, Latino, and Asian adolescents, in addition to those living in households where English is not the primary language, are more likely to report poor visual function than their non-Hispanic White counterparts [28,29,30,31]. Parental barriers also play a role, with parental lack of awareness of school vision screening results contributing to inconsistent follow-up care following a failed screening [31,32,33]. Several of the sociodemographic barriers to adolescent visual health are also posited to be linked to parental education levels, with children of parents with less than a high school education having higher odds of vision difficulty than children whose parents completed a professional or doctoral degree [12].

The objective of this retrospective cross-sectional study is to explore how caregiver social determinants of health, appraisal of healthcare provider effectiveness, and insurance coverage influence caregiver ability to have their adolescent child access vision care, including completion of annual vision screening, visiting an ophthalmologist or optometrist, and completion of recommended additional screenings. Our results will inform the development of future evidence-based educational interventions to raise awareness on the importance of annual vision screenings in US adolescents and emphasize the need for screening mandates to advocate for this important public health issue.

## 2. Methods

### 2.1. Data, Setting, and Population

The National Survey of Children’s Health (NSCH) is a household survey conducted by the U.S. Department of Health and Human Services Health Resources and Services Administration’s Maternal and Child Health Bureau, on various aspects related to the physical and emotional health, quality of health care, and the child’s family, as well as neighborhood, school, and social context, of children aged 0–17 years old in the United States and has been collected annually since 2016 [34,35,36]. Households are contacted by mail to identify those with one or more children, upon which one child is randomly selected to participate in the survey. The selected child is the subject of a follow-up topical questionnaire, which is then completed by an adult who is familiar with the child’s health. Surveys were offered in English and Spanish and could be completed online, on paper, or over the phone with a telephone questionnaire assistance (TQA) agent. One of three age-based topical questionnaires is provided based on the sample child’s age: 0 through 5 years old, 6 through 11 years old, and 12 through 17 years old. We used NSCH data for 12–17-year-old adolescents for the years 2022 and 2023 (n = 37,425). The overall survey response rate for each study year was 39.1% in 2022 and 35.8% in 2023. We decided to focus this study on the 12–17-year-old age range due to evidence from research studies of a decline in vision testing as adolescents aged, with older teens significantly less likely to have undergone recent vision assessments both during and post-COVID-19 pandemic [8,37,38]. This paper was deemed exempt from the Florida Atlantic University IRB review (IRB#2505164) based on the requirements delineated in the Declaration of Helsinki because it consists of secondary data analysis from a public database.

### 2.2. Outcome Variables

All our outcome measures were based on survey items that asked parents to report on whether their child has received a vision screening or exam within the past two years. We generated three variables measuring the parent-reported prevalence of adolescent vision screenings for adolescents aged 12–17 years old: Visited Ophthalmologist or Optometrist, Completion of Vision Screening, and Additional Vision Screening Recommended. Responses for each of these variables were collected based on the four questions asked in section C. of the NSCH survey. Survey items consisted of “*DURING THE PAST 2 YEARS, has this child received a vision screening from a care provider other than an eye doctor?*” with a binary response (Yes/No). Subsequently, the survey asks, “*If yes, was it recommended that this child see an eye doctor or other eye care provider for an eye examination or additional vision services as a result of the vision screening?*” with a similar binary response (Yes/No). The second binary question consists of “*DURING THE PAST 2 YEARS, has this child seen an eye doctor?*”, and subsequently followed by “*If yes, what care has this child received from the eye doctor?*” with a four option categorical response (“*Received eye examination*”*;* “*Prescribed eyeglasses or contact lenses*”*;* “*Diagnosis of a vision disorder other than nearsighted, farsighted, or astigmatism*”*;* “*Some other care*”). The term “eye doctor” explicitly includes both optometrists and ophthalmologists, as clarified in the NSCH survey (Supplementary File S1).

### 2.3. Covariates

Multiple studies have identified differences in caregiver sociodemographic characteristics and social determinants of health and their relative impact as covariates on the completion of adolescent vision screening in the United States [39,40]. We considered the following adolescent primary caregiver characteristics: Sex (binary; male and female), Age in years (categorical; grouped into four age brackets: 18–25, 26–35, 36–50, 51–75), Birth inside or outside the United States (binary), Highest level of completed schooling (categorical; 8th grade or less; 9th–12th grade No diploma; High School Graduate or GED Completed; Completed a vocational, trade, or business school program; Some College Credit, but No Degree; Associate Degree (AA, AS); Bachelor’s Degree (BA, BS, AB); Master’s Degree (MA, MS, MSW, MBA); Doctorate (PhD, EdD) or Professional Degree (MD, DDS, DVM, JD)); Marital status (categorical: Married; Never Married, living with partner; Never Married; Divorced; Separated; Widowed); and employment status (categorical: Employed full-time; Employed part-time; Working without pay; Not employed but looking for work; Not employed and not looking for work; Retired).

### 2.4. Independent Variables

We tested caregiver appraisal of insurance coverage and the quality of provided care received by their child’s doctor to determine their impact upon an adolescent receiving vision care. We considered whether the primary caregiver had their child on an insurance that meets needs, allows them to see needed providers, and has affordable out of pocket costs. Each of these three categorical variables were measured on a 4-Likert scale ranging from “Always”–“Never”. Six categorical characteristics of their doctor’s quality of care were assessed: “Doctor Spends Enough Time with Patient”, “Doctor Listened Carefully to Patient”, “Doctor’s Sensitivity to Patient’s Values”, “Doctor Provided Important Information”, “Doctor Made Care Feel Like A Partnership”, “Doctor’s Communication Effectiveness”, with the first five being a scale of “Always”, “Usually”, “Sometimes or Never”, and the last being a scale of “Very satisfied”, “Somewhat satisfied”, “Somewhat dissatisfied or very dissatisfied”.

### 2.5. Analysis

Data analysis was carried out with IBM SPSS Statistics (version 29). First, we selected responses for adolescents aged 12–17 years old, inclusive. For the outcome variables, the answers were coded as follows: “yes” = 1, “no” = 2, logical skip = 2, all other responses = systems missing. “Additional Vision Screening Recommended” was a new, recoded variable based off of an adolescent receiving a vision screening in the past two years from a care provider other than doctor and then also being recommended that the child see an eye doctor for additional screening. For the covariates, they were coded numerically in the order they were listed, e.g., Highest level of completed schooling had “8th grade or less” = 1; “9th–12th grade No diploma” = 2; “High School Graduate or GED Completed” = 3, etc. Independent Variables were likewise coded numerically, with “Always” = 1, “Usually” = 2, “Sometimes” = 3, “Never” = 4; and “Very satisfied” = 1, “Somewhat satisfied” = 2, “Somewhat dissatisfied or very dissatisfied” = 3. We generated descriptive summary statistics for the selected sample by computing numbers and percentages for our dependent variables and our covariate variables. Next, binary logistic regression of the dependent and independent variables was performed, calculating OR, 95% CI, and *p*-values. We then re-ran binary logistic regression for the recoded additional screening recommended outcome variable and independent variables consisting of each of the quality of care received by the child’s provider and type of insurance coverage. Regression analysis controlled for child’s caregiver characteristics as covariates including sex, age in years, birth in the United States, highest level of schooling attained, marital status, and employment status.

## 3. Results

### 3.1. Adolescent Completion of Eye Screenings for Ages 12–17

The majority (60.4%) of the 37,212 survey respondents reported their child seeing an eye doctor within the past 2 years, while 59.0% had received a vision screening from a provider other than a doctor. Of those who had seen an ophthalmologist or optometrist, 89.3% received an eye examination from the physician. Among the participants who received an eye examination, 63.8% were prescribed eyeglasses or contact lenses, while 8.7% (1972) received a diagnosis other than myopia, hyperopia, or astigmatism. For participants whose children completed their vision screenings at locations other than an eye doctor in the past two years, only 29.8% were recommended for referral for eye examination or additional services as the result of their screening (Table 1).

### 3.2. Adolescent Caregiver Sample Characteristics

A total of 37,212 caregivers participated in the survey. More than half of these respondents were female (66.2%) and in the age range of 36–50 years old (63.04%). The majority were born in the United States (80.5%) and were married (71.5%). Of the 17,478 participants who answered the question regarding employment, 71.5% were employed full-time while 11.5% were working part-time, 4.4% were retired, and 7.5% indicated they were unemployed and not looking for work. More than half (64%) of all caregivers had a college degree (including an Associate’s, Bachelor’s, Master’s, Doctorate, or Professional degree) while 16.7% had a high school degree or less (Table 2).

### 3.3. Binary Logistic Regression

A series of binary logistic regression was carried out to examine associations between the six criteria of the caregiver’s appraisal of child health care provider’s effectiveness (3-Likert Scale; Always-Sometimes or Never) and each of the three adolescent vision care variables (completion of vision screening, visited ophthalmologist/optometrist, and additional vision screening recommended). The responses “Sometimes” and “Never” were combined to avoid small cell sizes for the purpose of analysis. A second series of binary logistic regression explored associations between the three criteria of insurance coverage (4-Likert scale) and two criteria of adolescent vision care (completion of vision screening, visited ophthalmologist/optometrist in the past two years).

### 3.4. Caregiver’s Appraisal of Child Health Care Provider Effectiveness and Its Influential Role on Adolescent Vision Screening

Significant associations were reported across five of six criteria of healthcare provider effectiveness (Table 3). Compared to caregivers who reported their doctor “Always” spends enough time with them, those who responded that they “Sometimes or Never” spent enough time with the patient were significantly less likely to complete a vision screening (OR = 1.22; 95% CI = 1.063–1.401; *p* = 0.005). Similarly, adolescents whose caregivers reported that the doctor “Usually” (OR = 1.156; 95% CI = 1.055–1.267; *p* = 0.002) or “Sometimes or Never” (OR = 1.371; 95% CI = 1.193–1.576; *p* = <.001) spends enough time with them had higher odds of not visiting an ophthalmologist or optometrist in the past 24 months.

Adolescents whose caregivers felt the doctor “usually” listened carefully to the patient were more likely to not complete a vision screening (OR = 1.216; 95% CI = 1.085–1.362; *p* = <0.001) and had 0.868 times the odds of not visiting an ophthalmologist (OR = 0.868; 95% CI = 0.774–0.973; *p* = 0.015) compared to those who felt the doctor always listened to the patient. Those whose caregivers reported they “Sometimes or Never” listened carefully were significantly less likely to complete a vision screening (OR = 1.380; 95% CI = 1.118–1.703; *p* = 0.003).

Compared to caregivers who “always” had the adolescent healthcare provider provide them with important information, those who responded “usually” had 0.868 times the odds of completing a vision screening in the past 24 months (*p* = 0.016). Moreover, caregivers who felt the doctor “Sometimes or Never” provided important information had 0.738 times the odds of having their adolescent complete a vision screening (*p* = 0.003).

Furthermore, caregivers who responded “usually” to having a healthcare provider who makes care feel like a joint partnership were 1.173 times (*p* = 0.006) less likely to visit an eye doctor in the past 24 months. Similarly, those who selected “sometimes or never” for this question were 1.239 times (*p* = 0.030) less likely to visit an eye doctor. Finally, compared to those who were “very satisfied” with the adolescent healthcare provider’s communication effectiveness, those who responded “somewhat dissatisfied or very dissatisfied” were 0.642 times (*p* = 0.002) as likely to have an additional vision screening recommended to them in the past 24 months.

### 3.5. Caregiver’s Appraisal of Child Health Insurance Coverage and Its Influential Role on Adolescent Vision Screening

Significant associations were reported across two of three criteria of insurance coverage (Table 4). Compared to caregivers who reported “always” having insurance coverage that met their needs, those who selected “sometimes” had 1.160 times the odds of not completing a vision screening for their adolescent (*p* = 0.010) and 1.251 times the odds of not having visited an eye doctor (*p* < 0.001). Similarly, those who selected “never” having an insurance that met their needs had 1.516 times the odds of not completing a vision screening for their adolescent (*p* < 0.001) and 1.298 times the odds of not having visited an eye doctor (*p* = 0.030). Caregivers who “usually” deemed their out-of-pocket costs reasonable were 0.9 times (*p* = 0.002) less likely to visit an eye doctor in the past 24 months. Similarly, those who selected “sometimes” for this question were 0.846 (*p* < 0.001) times less likely to visit an eye doctor. Additionally, those who selected “never” for this question were 0.807 (*p* < 0.001) times less likely to visit an eye doctor. Insurance allowing the adolescent to see needed providers was not found to have significant associations within this dataset.

## 4. Discussion

The present study examines factors that impact parents’ ability to obtain yearly vision exams for their adolescent children. In order to address this pressing public health issue, our findings will guide initiatives to educate key stakeholders on the need for annual vision screenings for adolescents and implement screening mandates.

We identified in our analysis remarkably high prevalence rates for prescriptions required in adolescents for eyeglasses or contact lenses (63.8%). However, only 29.8% of adolescents were recommended for referral for eye examination or additional services as the result of their screening. Myopia is the most common refractive error in children, and the pooled prevalence of childhood myopia worldwide is estimated to have increased from 24.32% in 1990 to 35.81% in 2023 [41,42]. The American Academy of Ophthalmology (AAO) reports that upwards of 9.2% of children aged 5–17 years in the United States have myopia. Despite 60.4% of adolescents in our sample receiving an eye exam from a doctor in the past 2 years, reported gaps in annual eye screenings remain. A review of state vision screen mandates in 2021 found that 41 states require vision screening for school-aged children in elementary school but only 30 states and 19 states for middle school and high school, respectively [43]. The COVID-19 pandemic has likely worsened this issue as 23.7% of states waived their vision screening requirements during the 2020–2021 academic year [43]. Furthermore, children who identify as a racial/ethnic minority (Hispanic, non-Hispanic Asian, or non-Hispanic Black), reside in low-income households, or reside in households where English is not the primary language have lower odds of receiving vision screening and specialist care [31]. Caregivers most frequently identify cost, difficulties making an appointment, and concerns related to eligibility as the largest barriers for obtaining pediatric vision services [44]. Consequently, use of vision screening and follow-up care services remains a significant issue among adolescents.

Our study also found significant associations between vision screening completion and five of six criteria of healthcare provider effectiveness (listening, spending sufficient time, providing important information, joint partnership, and communication effectiveness). These findings reflect recommendations from the AAO that vision care practitioners communicate effectively with patients, listen closely to their unique needs, and educate them on their conditions in order to ensure patient involvement and adherence to the treatment plan [6]. In a survey of parents whose children failed vision screening tests, more than half of parents who did not obtain follow-up reported that they were not informed of the screening results [32]. Highlighting the need for appropriate health education, over 25% of parents in that survey did not identify follow-up eye care for their child as a high priority and nearly 30% reported not having information sources related to eye diseases [32]. Likewise, a qualitative study in England identified parental perceived severity of visual deficits as the major determinant in utilization of screening and follow-up [45]. Collectively, our findings and those from existing literature emphasize the role of effective patient-provider communication in improving vision screening completion.

Our analysis further identified significant patterns among caregivers regarding the adequacy of their insurance coverage and the resulting adolescent vision care habits, with responses indicating poorer insurance coverage significantly increasing the odds of having not engaged in important vision care habits such as completing an adolescent vision screening or visiting an ophthalmologist or optometrist. These results echo the findings of multiple large-scale studies that have demonstrated that uninsured adolescents, or those with gaps in insurance coverage, are significantly less likely to receive routine vision testing and are at markedly higher risk for unmet vision care needs compared to their insured peers [8,27,46]. The downstream effects of such led these uninsured adolescents to experience higher rates of uncorrected refractive error, visual impairment, and associated academic and psychosocial consequences [24,28]. In this context, the criticality of adequate insurance coverage is highlighted as it not only facilitates access to preventive vision screening and timely diagnosis of ocular conditions but also improves the likelihood of receiving necessary refractive correction (e.g., glasses), which is often unaffordable for uninsured families [24,28]. This criticality is evidenced in the work done by Muhammad and Tumin, which revealed that gaps in insurance coverage are associated with nearly 19-fold higher odds of unmet vision care needs [27]. These disparities further extend to overall health, in that uninsured adolescents are more likely to lack a usual source of care, have unmet health needs, and forgo physician contact, which can exacerbate both physical and mental health concerns [24].

To address healthcare challenges in adolescent eyecare, there have been several proposed programs, community interventions, and policies aimed toward improving care for socioeconomically disadvantaged adolescents. School-based programs have demonstrated substantial potential in addressing some of these disparities through improved uptake and adherence, especially when paired with the provision of free spectacles [8]. For instance, an evaluation of a combined school-based screening and mobile clinic program in Virginia showed that in the 2019–2020 school year, 14,006 students from 58 schools were screened, while 4238 (30.3%) were referred, out of which the percentage of economically disadvantaged students was positively correlated to referral rate [47]. Additionally, between 2019 and 2020, the mobile clinic examined 3095 students from the same 58 schools, out of which 72.9% were prescribed glasses and 16.3% were referred for a more comprehensive eye examination and treatment. Hence, a combined screening program and mobile clinic traveling directly to schools can act as a pipeline for delivering eye care to at-risk children and adolescents [47]. The American Academy of Pediatrics has hosted multiple committees on the matter of socioeconomic healthcare disparities, in which they have advocated for the full implementation of Medicaid’s Early and Periodic Screening, Diagnostic, and Treatment (EPSDT) benefit, permanent funding for the Children’s Health Insurance Program (CHIP), and for policies that minimize enrollment barriers and coverage disruptions. These measures are designed to address insurance gaps that disproportionately affect low-income and minority families, thereby aiming to reduce disparities in access to preventive and therapeutic eye care [48,49]. In pursuit of improving these disparities, a holistic approach that involves primary caregivers and factors in social determinants of health is all but essential. Caregivers’ involvement and awareness directly influence adolescents’ receipt of vision testing and follow-up care, and social determinants such as income, education, and insurance status are consistently linked to disparities in both eye health and overall health outcomes [8,50].

## 5. Limitations

This study has several important limitations that should be considered when interpreting the findings. First, all data are based on parental self-report, including whether the adolescent received a vision screening, the nature of care received from eye care providers, perceptions of provider communication quality, and satisfaction with insurance coverage. These responses are susceptible to recall bias, particularly regarding events over a two-year period, as well as social desirability bias, which may lead caregivers to over report positive health behaviors or care experiences. Second, the cross-sectional nature of the NSCH limits our ability to draw causal inferences. Although the NSCH is collected annually, it is not longitudinal with respect to following the same children over time; thus, we cannot assess temporal trends or within-subject changes in vision care utilization. Third, the survey does not include information on affording standard equipment for vision care (eyeglasses/contacts) after having an annual eye checkup and receiving prescription. Oftentimes the out-of-pocket expenses for prescription contacts for myopia corrections could be expensive and may be limiting to follow doctor prescribed corrective actions. Additionally, the survey’s response rates (39.1% in 2022 and 35.8% in 2023) raise concerns about potential nonresponse bias, as families who choose to participate may differ in meaningful ways from those who do not. Finally, the survey does not capture data from adolescents directly, which limits our understanding of their personal experiences, attitudes, or potential barriers to vision care that may not be fully reflected through caregiver reports.

## 6. Conclusions

In conclusion, this study underscores the substantial impact of eye care doctor effectiveness, insurance adequacy, and related socioeconomic factors on adolescent vision care engagement. The association between inadequate insurance coverage and decreased likelihood of receiving routine vision screenings or ophthalmologic or optometrist evaluation highlights a critical barrier to improving eye health for adolescents. Given the well-established links between uncorrected visual impairment and negative academic and psychosocial outcomes, targeted public health responses are crucially needed. These responses must go beyond expanding access to vision services alone through adopting a comprehensive, family-centered framework that considers social determinants of health and empowers primary caregivers. Sustainable strategies could include policy-level efforts to ensure continuous and comprehensive insurance coverage, increased investment in school-based screening programs, and integrated caregiver education on the importance of adolescent vision care. Ultimately, meaningful improvements in adolescent vision health will necessitate a coordinated, multilevel approach that prioritizes access, education, and the involvement of the family unit as a whole.

## Figures and Tables

**Table 1:** Sample Frequencies and Percentages of Adolescents Aged 12–17 Who Completed Eye Screenings.

Adolescent Completion of Eye Screenings for Ages 12–17 (N = 37,212)	Count (n=)	Percentage (%)
Adolescent who saw an eye doctor ever (0–5 years)/during the past 2 years		
Yes	22,601	60.4
No	14,611	39.0
Received eye examination from an eye doctor ever (0–5 years)/during the past 2 years		
Yes	20,178	89.3
No	2211	9.8
Received prescription for eyeglasses or contact lenses from an eye doctor ever (0–5 years)/during the past 2 years		
Yes	14,422	63.8
No	7967	35.3
Children who were diagnosed to have a vision disorder other than nearsighted, farsighted, or astigmatism by an eye doctor even (0–5 years)/during the past 2 years		
Yes	1972	8.7
No	20,417	91.3
Adolescent who received a vision screening from a provider other than an eye doctor ever (0–5 years)/during the past 2 years (12–17 years)		
*Yes*	22,084	59.0
*No*	15,205	40.6
Recommended for an eye examination or additional vision service as a result of the vision screening		
Yes	6589	29.8
No	15,495	70.2

**Table 2:** Sample Adolescent Caregiver Characteristics.

Child’s Caregiver Characteristics	Count (n=)	Percentage (%)
Caregiver Sex (N = 36,313)		
Male	11,543	30.8
Female	24,770	66.2
Caregiver Age (N = 35,957)		
18–25	122	0.33
26–35	1733	4.82
36–50	22,667	63.04
51–75	11,435	31.80
Caregiver’s Birth Location (N = 36,313)		
In the United States	30,123	80.5
Outside of the United States	6190	16.5
Caregiver’s Highest Level of Completed Education (N = 37,425)		
8th grade or less	567	1.5
9th–12th grade; No diploma	1017	2.7
High School Graduate or GED Completed	4675	12.5
Completed a vocational, trade, or business school program	1939	5.2
Some College Credit, but No Degree	5298	14.2
Associate Degree (AA, AS)	3690	9.9
Bachelor’s Degree (BA, BS, AB)	10,929	29.2
Master’s Degree (MA, MS, MSW, MBA)	7057	18.9
Doctorate (PhD, EdD) or Professional Degree (MD, DDS, DVM, JD)	2253	6.0
Caregiver Marital Status (N = 36,113)		
Married	26,767	71.5
Never Married, living with partner	1529	4.1
Never Married	1930	5.2
Divorced	4280	11.4
Separated	781	2.1
Widowed	826	2.2
Caregiver Employment Status (N = 17,478)		
Employed full-time	12,515	71.6
Employed part-time	2004	11.5
Working WITHOUT pay	185	1.1
Not employed but looking for work	679	3.9
Not employed and not looking for work	1318	7.5
Retired	777	4.4

**Table 3:** Binary logistic regression of the Association Between Caregiver’s Appraisal of Healthcare Provider’s Effectiveness and Three Adolescent Vision Care Criteria.

Caregiver’s Appraisal of Healthcare Provider’s Effectiveness	Adolescent Vision Care														
	Completion of Vision Screening					Visited Ophthalmologist/Optometrist					Additional Vision Screening Recommended				
	Yes	No	OR	95% CI	*p*-Value	Yes	No	OR	95% CI	*p*-Value	Yes	No	OR	95% CI	*p*-Value
**Doctor Spends Enough Time with Patient**															
**Always**	11,937	6556	Ref.	Ref.	Ref.	11,668	6793	Ref.	Ref.	Ref.	8643	3294	Ref.	Ref.	Ref.
**Usually**	5343	3264	1.010	0.922–1.108	0.824	5370	3225	1.156	1.055–1.267	0.002 *	3672	1671	0.897	0.756–1.064	0.212
**Sometimes or Never**	1416	1089	1.220	1.063–1.401	0.005 *	1482	1014	1.371	1.193–1.576	<0.001 *	870	546	0.810	0.621–1.058	0.122
**Doctor Listened Carefully to Patient**															
**Always**	13,405	7353	Ref.	Ref.	Ref.	12,968	7747	Ref.	Ref.	Ref.	9649	3756	Ref.	Ref.	Ref.
**Usually**	4465	2290	1.216	1.085–1.362	<0.001 *	4663	2715	0.868	0.774–0.973	0.015 *	3037	1428	0.916	0.737–1.139	0.430
**Sometimes or Never**	786	612	1.380	1.118–1.703	0.003 *	845	551	0.868	0.701–1.075	0.194	473	313	0.778	0.520–1.163	0.221
**Doctor’s Sensitivity to Patient’s Needs**															
**Always**	13,941	7732	Ref.	Ref.	Ref.	13,578	8052	Ref.	Ref.	Ref.	10,031	3910	Ref.	Ref.	Ref.
**Usually**	3886	2533	1.044	0.935–1.165	0.445	4007	2409	0.975	0.872–1.089	0.653	2627	1259	0.939	0.762–1.158	0.558
**Sometimes or Never**	802	601	1.085	0.895–1.315	0.405	870	532	0.884	0.727–1.076	0.219	482	320	0.892	0.616–1.292	0.545
**Doctor Provided Important Information**															
**Always**	13,682	7647	Ref.	Ref.	Ref.	13,362	7921	Ref.	Ref.	Ref.	9832	3850	Ref.	Ref.	Ref.
**Usually**	4141	2673	0.868	0.774–0.974	0.016 *	4251	2556	0.951	0.847–1.068	0.395	2835	1306	1.094	0.877–1.364	0.427
**Sometimes or Never**	821	566	0.738	0.602–0.905	0.003 *	856	530	0.937	0.764–1.150	0.535	487	334	0.964	0.659–1.409	0.849
**Doctor Made Care Feel Like Partnership**															
**Always**	13,781	7632	Ref.	Ref.	Ref.	13,642	7911	Ref.	Ref.	Ref.	9849	3932	Ref.	Ref.	Ref.
**Usually**	3948	2580	1.112	0.994–1.243	0.064	4042	2473	1.173	1.048–1.313	0.006 *	2716	1232	1.022	0.826–1.265	0.842
**Sometimes or Never**	946	688	1.061	0.876–1.285	0.547	999	635	1.239	1.021–1.503	0.030 *	602	344	1.636	1.131–2.368	0.009 *
**Doctor’s Communication Effectiveness**															
**Very Satisfied**	8891	4951	Ref.	Ref.	Ref.	8946	4855	Ref.	Ref.	Ref.	6238	2653	Ref.	Ref.	Ref.
**Somewhat Satisfied**	3416	2040	0.952	0.881–1.028	0.208	3529	1928	0.953	0.882–1.031	0.230	2248	1168	0.916	0.793–1.058	0.231
**Somewhat Satisfied or Very Dissatisfied**	686	425	0.936	0.809–1.082	0.371	708	402	0.941	0.813–1.090	0.416	418	268	0.642	0.488–0.645	0.002 *

* Significance set at *p* < 0.05.

**Table 4:** Binary logistic regression of the Association Between Caregiver’s Appraisal of Insurance Coverage and Two Adolescent Vision Care Criteria.

Caregiver’s Appraisal of Insurance Coverage	Adolescent Vision Care									
	Completion of Vision Screening					Visited Ophthalmologist/Optometrist				
	Yes	No	OR	95%	*p*-Value	Yes	No	OR	95%	*p*-Value
**Insurance Meets Needs**										
**Always**	12,997	8728	Ref.	Ref.	Ref.	13,324	8353	Ref.	Ref.	Ref.
**Usually**	6637	4334	1.003	0.944–1.066	0.930	6769	4186	1.037	0.975–1.102	0.250
**Sometimes**	1259	927	1.160	1.036–1.298	0.010 *	1251	932	1.251	1.118–1.401	<0.001 *
**Never**	180	203	1.516	1.199–1.917	<0.001 *	203	179	1.298	1.025–1.644	0.030 *
**Insurance Allows To See Needed Providers**										
**Always**	15,172	10,165	Ref.	Ref.	Ref.	15,562	9716	Ref.	Ref.	Ref.
**Usually**	4966	3313	1.012	0.949–1.081	0.709	5062	3213	1.038	0.972–1.108	0.271
**Sometimes**	846	601	0.992	0.870–1.131	0.905	832	615	1.096	0.961–1.249	0.173
**Never**	87	108	1.363	0.984–1.889	0.063	98	94	1.251	0.901–1.736	0.181
**Out Of Pocket Costs Were Deemed Reasonable**										
**Always**	3435	2252	Ref.	Ref.	Ref.	3450	2227	Ref.	Ref.	Ref.
**Usually**	6883	4539	1.001	0.937–1.070	0.975	7159	4246	0.900	0.842–0.963	0.002 *
**Sometimes**	4781	3048	0.941	0.874–1.014	0.110	4932	2879	0.846	0.785–0.912	<0.001 *
**Never**	1748	1128	0.921	0.836–1.015	0.097	1818	1054	0.807	0.731–0.890	<0.001 *

* Significance set at *p* < 0.05.

## Data Availability

The authors used data from a national public dataset, the National Survey of Children’s Health for years 2022–2023, and can share the specific dataset used upon request.

## Supplementary Materials

The following supporting information (S1: NSCH 2023 Survey) can be downloaded at: https://www.mdpi.com/article/10.3390/pediatric17050088/s1, Supplementary File S1: National Survey of Children’s Health.
