# Supplementary material for: Caregiver Socio-Economic Factors and Perceived Effectiveness of Care Delivery in Relation to US Adolescent Vision Care: A Retrospective Analysis from a National Database
Source: Pediatr Rep. 2025 Aug 31;17(5):88. doi: 10.3390/pediatric17050088 (PMC12452628; doi:10.3390/pediatric17050088)
Supplement: Supplementary file 1 [file pediatrrep-17-00088-s001.zip › pediatrrep-3803939-supplementary.pdf]

26023242

National Survey of Children's Health

A study by the U.S. Department of Health and Human Services to better understand the health issues faced by children in the United States today.

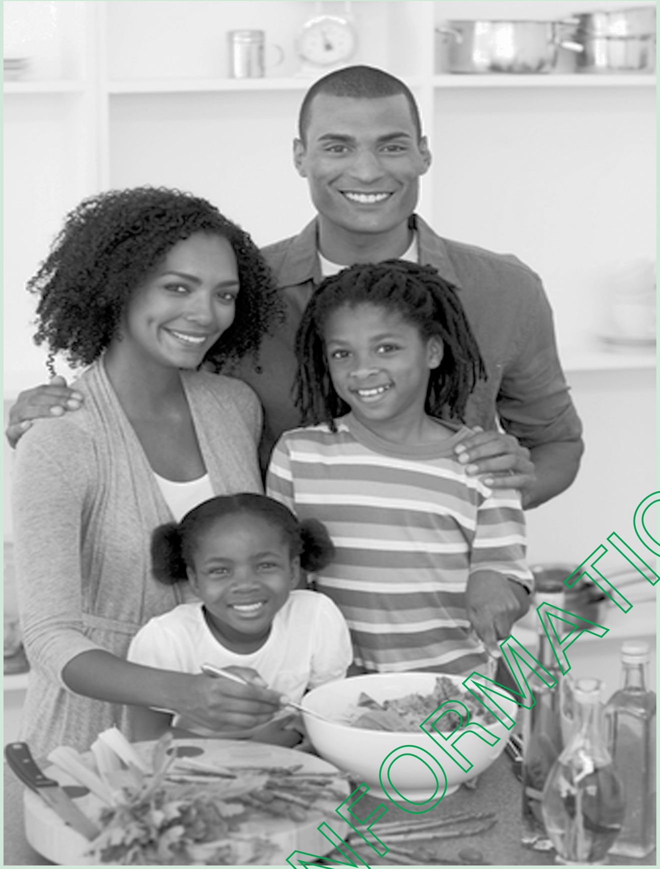

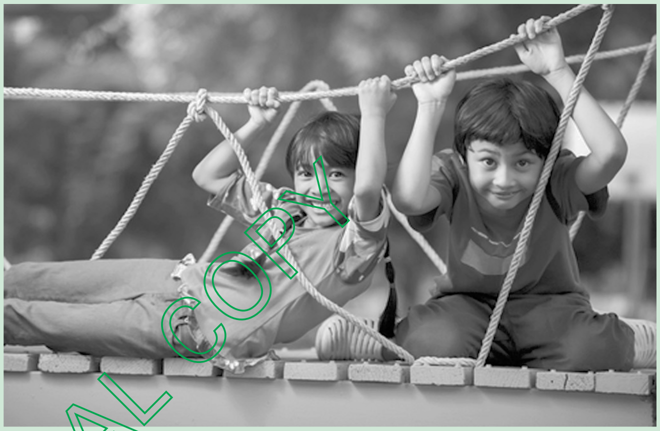

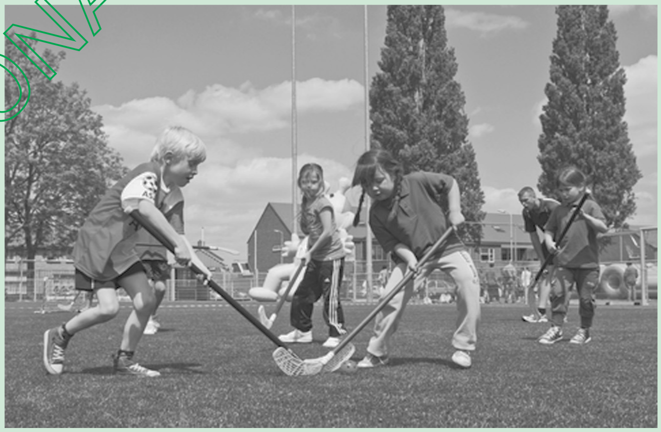

INFORMATIONAL COPY

The Census Bureau is required by law to protect your information. We are not permitted to publicly release your responses in a way that could identify your household. The Census Bureau is conducting this survey under the authority of Title 13, United States Code (U.S.C.), Section 8(b) (13 U.S.C. § 8(b)) and Section 501(a)(2) of the Social Security Act (42 U.S.C. § 701). Federal law protects your privacy and keeps your answers confidential under Title 13, U.S.C., Section 9 (13 U.S.C. § 9). Per the Federal Cybersecurity Enhancement Act of 2015, your data are protected from cybersecurity risks through screening of the systems that transmit your data.

Under the Privacy Act of 1974 (5 U.S.C. Section 552a), these records are maintained by the Census Bureau under SORN COMMERCE/CENSUS-3, Demographic Survey Collection (Census Bureau Sampling Frame). Access to records maintained in the system is restricted to Census Bureau employees and certain individuals authorized by Title 13, U.S. Code (designated as Special Sworn Status individuals). These individuals are subject to the same confidentiality requirements as regular Census Bureau employees.

Participation in this survey is voluntary and there are no penalties for refusing to answer questions. However, your cooperation in obtaining this much needed information is extremely important in order to ensure complete and accurate results.

NSCH-T2
(03/27/2023)

Start Here

Recently, you completed a survey that asked about the children usually living or staying at this address. Thank you for taking the time to complete that survey.

We now have some follow-up questions to ask about:

If the name listed above is not correct or does not correspond to a child living in this household, please call 1-800-845-8241 for assistance.

We have selected only one child per household in an effort to minimize the amount of time you will need to complete the follow-up questions.

The survey should be completed by a parent or adult caregiver who lives in this household and who is familiar with this child's health and health care.

Your participation is important. Thank you.

A. This Child's Health

A1 In general, how would you describe this child's health (the one named above)?

- ☐ Excellent
- ☐ Very good
- ☐ Good
- ☐ Fair
- ☐ Poor

A2 How would you describe the condition of this child's teeth?

- ☐ Excellent
- ☐ Very good
- ☐ Good
- ☐ Fair
- ☐ Poor

A3 DURING THE PAST 12 MONTHS, has this child had FREQUENT or CHRONIC difficulty with any of the following?

	Yes	No
**a.** Breathing or other respiratory problems (such as wheezing or shortness of breath)	☐	☐
**b.** Eating or swallowing because of a health condition	☐	☐
**c.** Digesting food, including stomach/intestinal problems, constipation, or diarrhea	☐	☐
**d.** Repeated or chronic physical pain, including headaches or other back or body pain	☐	☐
**e.** Toothaches	☐	☐
**f.** Bleeding gums	☐	☐
**g.** Decayed teeth or cavities	☐	☐

A4 Does this child have any of the following?

	Yes	No
**a.** Serious difficulty concentrating, remembering, or making decisions because of a physical, mental, or emotional condition	☐	☐
**b.** Serious difficulty walking or climbing stairs	☐	☐
**c.** Difficulty dressing or bathing	☐	☐
**d.** Deafness or problems with hearing	☐	☐
**e.** Blindness or problems with seeing, even when wearing glasses	☐	☐

Has a doctor or other health care provider EVER told you that this child has...

A5 Allergies (such as food, drug, insect, seasonal, or other)?

☐ Yes ☐ No

↳ **If yes, does this child CURRENTLY have the condition?**

☐ Yes ☐ No

↳ **If yes, is it:**

☐ Mild ☐ Moderate ☐ Severe

A6 Asthma?

☐ Yes ☐ No

↳ **If yes, does this child CURRENTLY have the condition?**

☐ Yes ☐ No

↳ **If yes, is it:**

☐ Mild ☐ Moderate ☐ Severe

INFORMATIONAL COPY

Has a doctor or other health care provider EVER told you that this child has...

A7 **Autoimmune disease (such as Type 1 Diabetes, Celiac, or Juvenile Idiopathic Arthritis)?**

☐ Yes ☐ No

↳ **If yes, is it:**

☐ Mild ☐ Moderate ☐ Severe

A8 **Cerebral Palsy?**

☐ Yes ☐ No

↳ **If yes, is it:**

☐ Mild ☐ Moderate ☐ Severe

A9 **Type 2 Diabetes?**

☐ Yes ☐ No

↳ **If yes, does this child CURRENTLY have the condition?**

☐ Yes ☐ No

↳ **If yes, is it:**

☐ Mild ☐ Moderate ☐ Severe

A10 **Epilepsy or Seizure Disorder?**

☐ Yes ☐ No

↳ **If yes, does this child CURRENTLY have the condition?**

☐ Yes ☐ No

↳ **If yes, is it:**

☐ Mild ☐ Moderate ☐ Severe

A11 **Heart Condition?**

☐ Yes ☐ No

↳ **If yes, was this child born with the condition?**

☐ Yes ☐ No

Does this child CURRENTLY have the condition?

☐ Yes ☐ No

↳ **If yes, is it:**

☐ Mild ☐ Moderate ☐ Severe

A12 **Frequent or severe headaches, including migraine?**

☐ Yes ☐ No

↳ **If yes, does this child CURRENTLY have the condition?**

☐ Yes ☐ No

↳ **If yes, is it:**

☐ Mild ☐ Moderate ☐ Severe

Has a doctor or other health care provider EVER told you that this child has...

A13 **Tourette Syndrome?**

☐ Yes ☐ No

↳ **If yes, does this child CURRENTLY have the condition?**

☐ Yes ☐ No

↳ **If yes, is it:**

☐ Mild ☐ Moderate ☐ Severe

A14 **Anxiety Problems?**

☐ Yes ☐ No

↳ **If yes, does this child CURRENTLY have these problems?**

☐ Yes ☐ No

↳ **If yes, is it:**

☐ Mild ☐ Moderate ☐ Severe

A15 **Depression?**

☐ Yes ☐ No

↳ **If yes, does this child CURRENTLY have the condition?**

☐ Yes ☐ No

↳ **If yes, is it:**

☐ Mild ☐ Moderate ☐ Severe

A16 **Down Syndrome?**

☐ Yes ☐ No

A17 **Blood Disorders (such as Sickle Cell Disease, Thalassemia, or Hemophilia)?**

☐ Yes ☐ No

↳ **If yes, is it:**

☐ Mild ☐ Moderate ☐ Severe

Was this child diagnosed with:

Sickle Cell Disease?	☐ Yes	☐ No
Thalassemia?	☐ Yes	☐ No
Hemophilia?	☐ Yes	☐ No
Other Blood Disorders?	☐ Yes	☐ No

Were any of these blood disorders identified through a blood test done shortly after birth?
These tests are sometimes called newborn screening.

☐ Yes ☐ No

INFORMATIONAL COPY

Has a doctor or other health care provider EVER told you that this child has...

A18 **Cystic Fibrosis?**

☐ Yes ☐ No

↳ **If yes, is it:**

☐ Mild ☐ Moderate ☐ Severe

Was this condition identified through a blood test done shortly after birth? *These tests are sometimes called newborn screening.*

☐ Yes ☐ No

A19 **Fetal Alcohol Spectrum Disorder (FASD)?**

☐ Yes ☐ No

Has a doctor, other health care provider, or educator EVER told you that this child has...
Examples of educators are teachers and school nurses.

A20 **Behavioral or Conduct Problems?**

☐ Yes ☐ No

↳ **If yes, does this child CURRENTLY have these problems?**

☐ Yes ☐ No

↳ **If yes, is it:**

☐ Mild ☐ Moderate ☐ Severe

A21 **Developmental Delay?**

☐ Yes ☐ No

↳ **If yes, does this child CURRENTLY have the condition?**

☐ Yes ☐ No

↳ **If yes, is it:**

☐ Mild ☐ Moderate ☐ Severe

Has a doctor, other health care provider, or educator EVER told you that this child has...
Examples of educators are teachers and school nurses.

A22 **Intellectual Disability (formerly known as Mental Retardation)?**

☐ Yes ☐ No

↳ **If yes, does this child CURRENTLY have the disability?**

☐ Yes ☐ No

↳ **If yes, is it:**

☐ Mild ☐ Moderate ☐ Severe

A23 **Speech or other language disorder?**

☐ Yes ☐ No

↳ **If yes, does this child CURRENTLY have the condition?**

☐ Yes ☐ No

↳ **If yes, is it:**

☐ Mild ☐ Moderate ☐ Severe

A24 **Learning Disability?**

☐ Yes ☐ No

↳ **If yes, does this child CURRENTLY have the disability?**

☐ Yes ☐ No

↳ **If yes, is it:**

☐ Mild ☐ Moderate ☐ Severe

A25 **Has a doctor or other health care provider EVER told you that this child has Autism or Autism Spectrum Disorder (ASD)?** *Include diagnoses of Asperger's Disorder or Pervasive Developmental Disorder (PDD).*

☐ Yes ☐ No → ***SKIP to question A30 on page 5***

↳ **If yes, does this child CURRENTLY have the condition?**

☐ Yes ☐ No

↳ **If yes, is it:**

☐ Mild ☐ Moderate ☐ Severe

A26 **How old was this child when a doctor or other health care provider FIRST told you that they had Autism, ASD, Asperger's Disorder or PDD?**

☐☐ Age in years ☐ Don't know

INFORMATIONAL COPY

A27 **What type of doctor or other health care provider was the FIRST to tell you that this child had Autism, ASD, Asperger's Disorder or PDD?**
Mark (X) ONE box.

- ☐ Primary Care Provider
- ☐ Specialist
- ☐ School Psychologist/Counselor
- ☐ Other Psychologist (Non-School)
- ☐ Psychiatrist
- ☐ Other, specify: ________
- ☐ Don't know

A28 **Is this child CURRENTLY taking medication for Autism, ASD, Asperger's Disorder or PDD?**

☐ Yes ☐ No

A29 **At any time DURING THE PAST 12 MONTHS, did this child receive behavioral treatment for Autism, ASD, Asperger's Disorder or PDD, such as training or an intervention that you or this child received to help with their behavior?**

☐ Yes ☐ No

A30 **Has a doctor or other health care provider EVER told you that this child has Attention Deficit Disorder or Attention-Deficit/Hyperactivity Disorder, that is, ADD or ADHD?**

☐ Yes ☐ No → ***SKIP to question* A33**

↳ **If yes, does this child CURRENTLY have the condition?**

☐ Yes ☐ No

↳ **If yes, is it:**

☐ Mild ☐ Moderate ☐ Severe

A31 **Is this child CURRENTLY taking medication for ADD or ADHD?**

☐ Yes ☐ No

A32 **At any time DURING THE PAST 12 MONTHS, did this child receive behavioral treatment for ADD or ADHD, such as training or an intervention that you or this child received to help with their behavior?**

☐ Yes ☐ No

A33 **Do you think this child has EVER had a concussion or brain injury?** *A concussion or brain injury is when a blow or jolt to the head causes problems such as headaches, dizziness, being dazed or confused, difficulty remembering or concentrating, vomiting, blurred vision, changes in mood or behavior, or being knocked out.*

☐ Yes ☐ No

↳ **If yes, did you seek medical care from a doctor or other health care provider?**

☐ Yes ☐ No

↳ **If yes, did a doctor or other health care provider tell you that your child had a concussion or brain injury?**

☐ Yes ☐ No

A34 **DURING THE PAST 12 MONTHS, how often have this child's health conditions or problems affected their ability to do things other children their age do?**

- ☐ This child does not have any health conditions → ***SKIP to question* B1**
- ☐ Never → ***SKIP to question* B1**
- ☐ Sometimes
- ☐ Usually
- ☐ Always

A35 **To what extent do this child's health conditions or problems affect their ability to do things?**

- ☐ Very little
- ☐ Somewhat
- ☐ A great deal

B. This Child as an Infant

B1 **Was this child born more than 3 weeks before their due date?**

- ☐ Yes
- ☐ No

B2 **What month and year was this child born?**
Birth Month / 4-Digit Birth Year

☐☐ / 2 0 ☐☐

B3 **How much did they weigh when born?** *Answer in pounds and ounces OR kilograms and grams. Your best estimate is fine.*

☐☐ pounds **AND** ☐☐ ounces

OR

☐☐ kilograms **AND** ☐☐☐ grams

INFORMATIONAL COPY

C. Health Care Services

C1 **DURING THE PAST 12 MONTHS, did this child see a doctor, nurse, or other health care professional for sick-child care, well-child check-ups, physical exams, hospitalizations or any other kind of medical care?** *Include health care visits done by video or phone.*

- ☐ Yes
- ☐ No ➔ ***SKIP to question C4***

C2 **If yes, DURING THE PAST 12 MONTHS, how many times did this child visit a doctor, nurse, or other health care professional to receive a PREVENTIVE check-up?** *A preventive check-up is when this child was not sick or injured, such as an annual or sports physical, or well-child visit.*

- ☐ 0 visits
- ☐ 1 visit
- ☐ 2 or more visits

C3 **Thinking about the LAST TIME you took this child for a PREVENTIVE check-up, about how long was the doctor or health care provider who examined this child in the room with you?** *Your best estimate is fine.*

- ☐ Less than 10 minutes
- ☐ 10-20 minutes
- ☐ More than 20 minutes

C4 **What is this child's CURRENT height?** *Your best estimate is fine.*

☐ feet **AND** ☐☐ inches

OR

☐ meters **AND** ☐☐☐ centimeters

C5 **How much does this child CURRENTLY weigh?** *Your best estimate is fine.*

☐☐☐ pounds

OR

☐☐ kilograms

C6 **Are you concerned about this child's weight?**

- ☐ Yes, it's too high
- ☐ Yes, it's too low
- ☐ No, I am not concerned

C7 **Has a doctor or other health care provider ever told you that this child is overweight?**

- ☐ Yes
- ☐ No

C8 **DURING THE PAST 12 MONTHS, did this child engage in any of the following?** *Mark (X) Yes or No for EACH item.*

	Yes	No
**a.** Skipping meals or fasting (Do NOT include skipping meals or fasting for religious reasons)	☐	☐
**b.** Having low interest in food	☐	☐
**c.** Extremely picky eating	☐	☐
**d.** Binge eating	☐	☐
**e.** Purging or vomiting after eating	☐	☐
**f.** Using diet pills, laxatives, or diuretics (water pills) to lose or maintain weight without a doctor's orders	☐	☐
**g.** Over-exercising	☐	☐
**h.** Not eating due to fear of vomiting or choking	☐	☐

INFORMATIONAL COPY

C9 ***Answer question C9 only if you marked "Yes" for at least one item in question C8. Otherwise skip to question C10.***

For question C9, consider only the behaviors you marked "Yes" to in question C8.

DURING THE PAST 12 MONTHS, how concerned were you about this child engaging in these behaviors?

- ☐ Very much
- ☐ Somewhat
- ☐ Not at all

C10 **DURING THE PAST 12 MONTHS, how concerned was this child about their weight, body shape, or body size?**

- ☐ Very much
- ☐ Somewhat
- ☐ Not at all

C11 **Is there a place you or another caregiver USUALLY take this child when they are sick or you need advice about their health?**

- ☐ Yes
- ☐ No ➔ ***SKIP to question C13 on page 7***

C12 If yes, where does this child USUALLY go first? *Mark (X) ONE box.*

- ☐ Doctor's Office
- ☐ Hospital Emergency Room
- ☐ Hospital Outpatient Department
- ☐ Urgent Care Center
- ☐ Clinic within a drug store or grocery store
- ☐ School (Nurse's Office, Athletic Trainer's Office)
- ☐ Other Clinic or Health Center
- ☐ Some other place

C13 Is there a place that this child USUALLY goes when they need routine preventive care, such as a physical examination or well-child check-up?

- ☐ Yes
- ☐ No → ***SKIP to question* C15**

C14 If yes, is this the same place this child goes when they are sick?

- ☐ Yes
- ☐ No

C15 DURING THE PAST 2 YEARS, has this child received a vision screening from a care provider other than an eye doctor? *The screening could have occurred at a pediatrician's office, in a school, preschool/child care center, or a community setting, using pictures, shapes, letters, or a camera like tool.*

- ☐ Yes ☐ No

↳ **If yes, was it recommended that this child see an eye doctor or other eye care provider for an eye examination or additional vision services as a result of the vision screening?** *An eye doctor may be referred to as an optometrist or ophthalmologist.*

- ☐ Yes ☐ No

C16 DURING THE PAST 2 YEARS, has this child seen an eye doctor? *An eye doctor may be referred to as an optometrist or ophthalmologist.*

- ☐ Yes ☐ No

↳ **If yes, what care has this child received from the eye doctor?** *Mark (X) ALL that apply.*

- ☐ Received eye examination
- ☐ Prescribed eyeglasses or contact lenses
- ☐ Diagnosis of a vision disorder other than nearsighted, farsighted, or astigmatism
- ☐ Some other care

C17 DURING THE PAST 12 MONTHS, did this child see a dentist or other oral health care provider for any kind of dental or oral health care? *Mark (X) ALL that apply.*

- ☐ Yes, saw a dentist
- ☐ Yes, saw other oral health care provider
- ☐ No → ***SKIP to question* C20**

C18 If yes, DURING THE PAST 12 MONTHS, did this child see a dentist or other oral health care provider for PREVENTIVE dental care, such as check-ups, dental cleanings, dental sealants, or fluoride treatments?

- ☐ No preventive visits in the past 12 months → ***SKIP to question* C20**
- ☐ Yes, 1 visit
- ☐ Yes, 2 or more visits

C19 If yes, DURING THE PAST 12 MONTHS, what PREVENTIVE dental service(s) did this child receive? *Mark (X) ALL that apply.*

- ☐ Check-up
- ☐ Cleaning
- ☐ Instruction on tooth brushing and oral health care
- ☐ X-Rays
- ☐ Fluoride treatment
- ☐ Sealant (plastic coatings on back teeth)
- ☐ Don't know

C20 DURING THE PAST 12 MONTHS, has this child received any treatment or counseling from a mental health professional? *Mental health professionals include psychiatrists, psychologists, psychiatric nurses, and clinical social workers.*

- ☐ Yes
- ☐ No, but this child needed to see a mental health professional
- ☐ No, this child did not need to see a mental health professional → ***SKIP to question* C22 *on page 8***

C21 How difficult was it to get the mental health treatment or counseling that this child needed?

- ☐ Not difficult
- ☐ Somewhat difficult
- ☐ Very difficult
- ☐ It was not possible to obtain care

INFORMATIONAL COPY

C22 **DURING THE PAST 12 MONTHS, has this child taken any medication because of difficulties with their emotions, concentration, or behavior?**

- ☐ Yes
- ☐ No

C23 **DURING THE PAST 12 MONTHS, did this child see a specialist other than a mental health professional?** *Specialists are doctors like surgeons, heart doctors, allergy doctors, skin doctors, and others who specialize in one area of health care.*

- ☐ Yes
- ☐ No, but this child needed to see a specialist
- ☐ No, this child did not need to see a specialist ➔ ***SKIP to question*** **C25**

C24 **How difficult was it to get the specialist care that this child needed?**

- ☐ Not difficult
- ☐ Somewhat difficult
- ☐ Very difficult
- ☐ It was not possible to obtain care

C25 **DURING THE PAST 12 MONTHS, was there any time when this child needed health care but it was not received?** *By health care, we mean medical care as well as other kinds of care like dental care, vision care, and mental health services.*

- ☐ Yes
- ☐ No ➔ ***SKIP to question*** **C28**

C26 **If yes, which types of care were not received?** *Mark (X) ALL that apply.*

- ☐ Medical Care
- ☐ Dental Care
- ☐ Vision Care
- ☐ Hearing Care
- ☐ Mental Health Services
- ☐ Other, specify: ________________

C27 **Did any of the following reasons contribute to this child not receiving needed health services?** *Mark (X) Yes or No for EACH item.*

	Yes	No
**a.** This child was not eligible for the services	☐	☐
**b.** The services this child needed were not available in your area	☐	☐
**c.** There were problems getting an appointment when this child needed one	☐	☐
**d.** There were problems with getting transportation or child care	☐	☐
**e.** The clinic or doctor's office wasn't open when this child needed care	☐	☐
**f.** There were issues related to cost	☐	☐

C28 **DURING THE PAST 12 MONTHS, how often were you frustrated in your efforts to get services for this child?**

- ☐ Never
- ☐ Sometimes
- ☐ Usually
- ☐ Always

C29 **DURING THE PAST 12 MONTHS, how many times did this child visit a hospital emergency room?** *Do NOT include visits to urgent care centers.*

- ☐ None
- ☐ 1 time
- ☐ 2-3 times
- ☐ 4 or more times

C30 **DURING THE PAST 12 MONTHS, was this child admitted to the hospital to stay for at least one night?**

- ☐ Yes
- ☐ No

C31 **Has this child EVER had a special education or early intervention plan?** *Children receiving these services often have an Individualized Family Service Plan (IFSP) or Individualized Education Plan (IEP).*

- ☐ Yes
- ☐ No ➔ ***SKIP to question*** **C34** ***on page 9***

INFORMATIONAL COPY

C32 **If yes, how old was this child at the time of the FIRST plan?**

☐☐ years **AND** ☐☐ months

C33 **Is this child CURRENTLY receiving services under one of these plans?**

- ☐ Yes
- ☐ No

C34 **Has this child EVER received special services to meet their developmental needs?** *Special services can include therapies such as speech, occupational, physical or behavioral or other services received to meet developmental needs.*

- ☐ Yes
- ☐ No → ***SKIP to question*** **C37**

C35 **If yes, how old was this child when they began receiving these special services?**

☐☐ years **AND** ☐☐ months

C36 **Is this child CURRENTLY receiving these special services?**

- ☐ Yes
- ☐ No

C37 **Has a doctor, other health care provider, or educator EVER recommended that this child be evaluated for a Fetal Alcohol Spectrum Disorder?**
Examples of educators are teachers and school nurses.

- ☐ Yes
- ☐ No
- ☐ Don't know

C38 **Has this child EVER received an evaluation for a Fetal Alcohol Spectrum Disorder?**

- ☐ Yes
- ☐ No
- ☐ Don't know

D. Experience with This Child's Health Care Providers

D1 **Do you have one or more persons you think of as this child's personal doctor or nurse?** *A personal doctor or nurse is a health professional who knows this child well and is familiar with this child's health history. This can be a general doctor, a pediatrician, a specialist doctor, a nurse practitioner, or a physician assistant.*

- ☐ Yes, one person
- ☐ Yes, more than one person
- ☐ No

D2 **DURING THE PAST 12 MONTHS, did this child need a referral to see any doctors or receive any services?**

- ☐ Yes
- ☐ No → ***SKIP to question*** **D4**

D3 **How difficult was it to get referrals?**

- ☐ Not difficult
- ☐ Somewhat difficult
- ☐ Very difficult
- ☐ It was not possible to get a referral

D4 ***Answer the following questions only if this child had a health care visit IN THE PAST 12 MONTHS. Otherwise skip to question*** **E1** ***on page 10.***

DURING THE PAST 12 MONTHS, how often did this child's doctors or other health care providers...

	Always	Usually	Sometimes	Never
**a.** Spend enough time with this child?	☐	☐	☐	☐
**b.** Listen carefully to you?	☐	☐	☐	☐
**c.** Show sensitivity to your family's values and customs?	☐	☐	☐	☐
**d.** Provide the specific information you needed concerning this child?	☐	☐	☐	☐
**e.** Help you feel like a partner in this child's care?	☐	☐	☐	☐

INFORMATIONAL COPY

D5 **DURING THE PAST 12 MONTHS, did you, another caregiver, or a health care provider need to make any decisions regarding this child's health care, such as whether to get prescriptions, referrals, or procedures?**

☐ Yes

☐ No → ***SKIP to question*** **D7**

D6 **If yes, DURING THE PAST 12 MONTHS, how often did this child's doctors or other health care providers...**

	Always	Usually	Sometimes	Never
a. Discuss with you the range of options to consider for their health care or treatment?	☐	☐	☐	☐
b. Make it easy for you to raise concerns or disagree with recommendations for this child's health care?	☐	☐	☐	☐
c. Work with you to decide together which health care and treatment choices would be best for this child?	☐	☐	☐	☐

D7 **DURING THE PAST 12 MONTHS, did anyone help you arrange or coordinate this child's care among the different doctors or services that this child uses?**

☐ Yes

☐ No

☐ Did not see more than one health care provider in the PAST 12 MONTHS → ***SKIP to question*** **D11**

D8 **DURING THE PAST 12 MONTHS, have you felt that you could have used extra help arranging or coordinating this child's care among the different health care providers or services?**

☐ Yes

☐ No → ***SKIP to question*** **D10**

D9 **If yes, DURING THE PAST 12 MONTHS, how often did you get as much help as you wanted with arranging or coordinating this child's health care?**

☐ Usually

☐ Sometimes

☐ Never

D10 **DURING THE PAST 12 MONTHS, how satisfied were you with the communication between this child's doctors and other health care providers?**

☐ Very satisfied

☐ Somewhat satisfied

☐ Somewhat dissatisfied

☐ Very dissatisfied

D11 **DURING THE PAST 12 MONTHS, did this child's health care provider communicate with the child's school, child care provider, or special education program?**

☐ Yes

☐ No → ***SKIP to question*** **E1**

☐ Did not need health care provider to communicate with these providers → ***SKIP to question*** **E1**

D12 **If yes, during this time, how satisfied were you with the health care provider's communication with the school, child care provider, or special education program?**

☐ Very satisfied

☐ Somewhat satisfied

☐ Somewhat dissatisfied

☐ Very dissatisfied

INFORMATIONAL COPY

E. This Child's Health Insurance Coverage

E1 **DURING THE PAST 12 MONTHS, was this child EVER covered by ANY kind of health insurance or health coverage plan?**

☐ Yes, this child was covered all 12 months → ***SKIP to question*** **E3** ***on page 11***

☐ Yes, but this child had a gap in coverage

☐ No

E2 **Is this child CURRENTLY covered by ANY kind of health insurance or health coverage plan?**

☐ Yes

☐ No → ***SKIP to question*** **F1** ***on page 11***

E3 **Is this child CURRENTLY covered by any of the following types of health insurance or health coverage plans?** *Mark (X) Yes or No for EACH item.*

	Yes	No
a. Insurance through a current or former employer or union	☐	☐
b. Insurance purchased directly from an insurance company	☐	☐
c. Medicaid, Medical Assistance, or any kind of government assistance plan for those with low incomes or a disability	☐	☐
d. TRICARE or other military health care	☐	☐
e. Indian Health Service	☐	☐
f. Other, specify:	☐	☐

E4 **How often does this child's health insurance offer benefits or cover services that meet this child's needs?**

- ☐ Always
- ☐ Usually
- ☐ Sometimes
- ☐ Never

E5 **How often does this child's health insurance allow them to see the health care providers they need?**

- ☐ Always
- ☐ Usually
- ☐ Sometimes
- ☐ Never

INFORMATIONAL COPY

F. Providing for This Child's Health

F1 **Including co-pays and amounts reimbursed from Health Savings Accounts (HSA) and Flexible Spending Accounts (FSA), how much money did you pay for this child's medical, health, dental, and vision care DURING THE PAST 12 MONTHS?** *Do not include health insurance premiums or costs that were or will be reimbursed by insurance or another source.*

- ☐ $0 (No medical or health-related expenses) → ***SKIP to question*** **F4**
- ☐ $1-$249
- ☐ $250-$499
- ☐ $500-$999
- ☐ $1,000-$5,000
- ☐ More than $5,000

F2 **How often are these costs reasonable?**

- ☐ Always
- ☐ Usually
- ☐ Sometimes
- ☐ Never

F3 **DURING THE PAST 12 MONTHS, did your family have problems paying for any of this child's medical or health care bills?**

- ☐ Yes
- ☐ No

F4 **DURING THE PAST 12 MONTHS, have you or other family members...**

	Yes	No
a. Left a job or taken a leave of absence because of this child's health or health conditions?	☐	☐
b. Cut down on the hours you work because of this child's health or health conditions?	☐	☐
c. Avoided changing jobs because of concerns about maintaining health insurance for this child?	☐	☐

F5 **IN AN AVERAGE WEEK, how many hours do you or other family members spend providing health care at home for this child?** *Care might include changing bandages, or giving medication and therapies when needed.*

- ☐ This child does not need health care provided at home on a weekly basis
- ☐ Less than 1 hour per week
- ☐ 1-4 hours per week
- ☐ 5-10 hours per week
- ☐ 11 or more hours per week

F6 **IN AN AVERAGE WEEK, how many hours do you or other family members spend arranging or coordinating health or medical care for this child, such as making appointments or locating services?**

- ☐ This child does not need health care coordinated on a weekly basis
- ☐ Less than 1 hour per week
- ☐ 1-4 hours per week
- ☐ 5-10 hours per week
- ☐ 11 or more hours per week

G. This Child's Schooling and Activities

G1 **DURING THE PAST 12 MONTHS, about how many days did this child miss school because of illness or injury?** *Include days missed from any formal home schooling.*

- ☐ No missed school days
- ☐ 1-3 days
- ☐ 4-6 days
- ☐ 7-10 days
- ☐ 11 or more days
- ☐ This child was not enrolled in school

G2 **DURING THE PAST 12 MONTHS, how many times has this child's school contacted you or another adult in your household about any problems they are having with school?**

- ☐ None
- ☐ 1 time
- ☐ 2 or more times

G3 **Across all subjects, what grades did this child get during the 2022-2023 school year?**

- ☐ Mostly A's
- ☐ Mostly A's and B's
- ☐ Mostly B's and C's
- ☐ Mostly C's and D's
- ☐ Mostly D's or lower
- ☐ This child's school does not give these grades

G4 **SINCE STARTING KINDERGARTEN, has this child repeated any grades?**

- ☐ Yes
- ☐ No

G5 **DURING THE PAST 12 MONTHS, did this child participate in...**

	Yes	No
a. A sports team or did they take sports lessons after school or on weekends?	☐	☐
b. Any clubs or organizations after school or on weekends?	☐	☐
c. Any other organized activities or lessons, such as music, dance, language, or other arts?	☐	☐
d. Any type of community service or volunteer work at school, place of worship, or in the community?	☐	☐
e. Any paid work, including regular jobs as well as babysitting, cutting grass, or other occasional work?	☐	☐

G6 **DURING THE PAST 12 MONTHS, how often did you attend events or activities that this child participated in?**

- ☐ Always
- ☐ Usually
- ☐ Sometimes
- ☐ Rarely
- ☐ Never

G7 **DURING THE PAST WEEK, on how many days did this child exercise, play a sport, or participate in physical activity for at least 60 minutes?**

- ☐ 0 days
- ☐ 1-3 days
- ☐ 4-6 days
- ☐ Every day

INFORMATIONAL COPY

G8 **Compared to other children their age, how much difficulty does this child have making or keeping friends?**

- ☐ No difficulty
- ☐ A little difficulty
- ☐ A lot of difficulty

G9 **DURING THE PAST 12 MONTHS, how often was this child bullied, picked on, or excluded by other children?** *Do not include siblings. If the frequency changed throughout the year, report the highest frequency.*

- ☐ Never (in the past 12 months)
- ☐ 1-2 times (in the past 12 months)
- ☐ 1-2 times per month
- ☐ 1-2 times per week
- ☐ Almost every day

G10 **DURING THE PAST 12 MONTHS, how often did this child bully others, pick on them, or exclude them?** *Do not include siblings. If the frequency changed throughout the year, report the highest frequency.*

- ☐ Never (in the past 12 months)
- ☐ 1-2 times (in the past 12 months)
- ☐ 1-2 times per month
- ☐ 1-2 times per week
- ☐ Almost every day

G11 **How often does this child...**

	Always	Usually	Sometimes	Never
**a.** Show interest and curiosity in learning new things?	☐	☐	☐	☐
**b.** Work to finish tasks they start?	☐	☐	☐	☐
**c.** Stay calm and in control when faced with a challenge?	☐	☐	☐	☐
**d.** Care about doing well in school?	☐	☐	☐	☐
**e.** Do all required homework?	☐	☐	☐	☐
**f.** Argue too much?	☐	☐	☐	☐

H. About You and This Child

H1 **Was this child born in the United States?**

- ☐ Yes → ***SKIP to question* H3**
- ☐ No

H2 **If no, how long has this child been living in the United States?**

☐☐ years **AND** ☐☐ months

H3 **How many times has this child moved to a new address since they were born?**

☐☐ Number of times

H4 **How often does this child go to bed at about the same time on weeknights?**

- ☐ Always
- ☐ Usually
- ☐ Sometimes
- ☐ Rarely
- ☐ Never

H5 **DURING THE PAST WEEK, how many hours of sleep did this child get on most weeknights?**

- ☐ Less than 6 hours
- ☐ 6 hours
- ☐ 7 hours
- ☐ 8 hours
- ☐ 9 hours
- ☐ 10 hours
- ☐ 11 or more hours

INFORMATIONAL COPY

H6 **ON MOST WEEKDAYS, about how much time did this child spend in front of a TV, computer, cellphone or other electronic device watching programs, playing games, accessing the internet or using social media?** *Do not include time spent doing schoolwork.*

- ☐ Less than 1 hour
- ☐ 1 hour
- ☐ 2 hours
- ☐ 3 hours
- ☐ 4 or more hours

H7 **How well can you and this child share ideas or talk about things that really matter?**

- ☐ Very well
- ☐ Somewhat well
- ☐ Not very well
- ☐ Not well at all

H8 **How well do you think you are handling the day-to-day demands of raising children?**

- ☐ Very well
- ☐ Somewhat well
- ☐ Not very well
- ☐ Not well at all

H9 **DURING THE PAST MONTH, how often have you felt...**

	Never	Rarely	Sometimes	Usually	Always
a. That this child is much harder to care for than most children their age?	☐	☐	☐	☐	☐
b. That this child does things that really bother you a lot?	☐	☐	☐	☐	☐
c. Angry with this child?	☐	☐	☐	☐	☐

H10 **DURING THE PAST 12 MONTHS, was there someone that you could turn to for day-to-day emotional support with parenting or raising children?**

- ☐ Yes
- ☐ No

I. About Your Family and Household

I1 **DURING THE PAST WEEK, on how many days did all the family members who live in the household eat a meal together?**

- ☐ 0 days
- ☐ 1-3 days
- ☐ 4-6 days
- ☐ Every day

I2 **Does anyone living in your household use cigarettes, cigars, or pipe tobacco?**

- ☐ Yes
- ☐ No → ***SKIP to question*** **I4**

I3 **If yes, does anyone smoke inside your home?**

- ☐ Yes
- ☐ No

I4 **Does anyone vape or use e-cigarettes inside your home?**

- ☐ Yes
- ☐ No

I5 **SINCE THIS CHILD WAS BORN, how often has it been very hard to cover the basics, like food or housing, on your family's income?**

- ☐ Never
- ☐ Rarely
- ☐ Somewhat often
- ☐ Very often

I6 **Which of these statements best describes your household's ability to afford the food you need DURING THE PAST 12 MONTHS?**

- ☐ We could always afford to eat good nutritious meals.
- ☐ We could always afford enough to eat but not always the kinds of food we should eat.
- ☐ Sometimes we could not afford enough to eat.
- ☐ Often we could not afford enough to eat.

INFORMATIONAL COPY

I7 At any time DURING THE PAST 12 MONTHS, even for one month, did anyone in your family receive...

	Yes	No
a. Cash assistance from a government welfare program?	☐	☐
b. Food Stamps or Supplemental Nutrition Assistance Program (SNAP) benefits?	☐	☐
c. Free or reduced-cost breakfasts or lunches at school?	☐	☐
d. School meal debit/Electronic Benefits Transfer (EBT) cards?	☐	☐
e. Benefits from the Women, Infants, and Children (WIC) Program?	☐	☐

I8 Does this child receive SSI, that is, Supplemental Security Income?
SSI is different from Social Security.

☐ Yes ☐ No

↳ **If yes, is this for a disability they have?**

☐ Yes ☐ No

I9 DURING THE PAST 12 MONTHS, was there a time when you were not able to pay the mortgage or rent on time?

☐ Yes
☐ No
☐ Don't know

I10 DURING THE PAST 12 MONTHS, how often were you worried or stressed about being evicted, foreclosed on, or having your housing condemned?

☐ Always
☐ Usually
☐ Sometimes
☐ Rarely
☐ Never

I11 DURING THE PAST 12 MONTHS, how many places has this child lived?

☐☐ Number of places

I12 SINCE THIS CHILD WAS BORN, have they ever been homeless or lived in a shelter? *Include living in a shelter, motel, temporary or transitional living situation, scattered site housing, or having no steady place to sleep at night.*

☐ Yes
☐ No
☐ Don't know

I13 In your neighborhood, is/are there...

	Yes	No
a. Sidewalks or walking paths?	☐	☐
b. A park or playground?	☐	☐
c. A recreation center, community center, or boys' and girls' club?	☐	☐
d. A library or bookmobile?	☐	☐
e. Litter or garbage on the street or sidewalk?	☐	☐
f. Poorly kept or rundown housing?	☐	☐
g. Vandalism such as broken windows or graffiti?	☐	☐

I14 To what extent do you agree with these statements about your neighborhood or community?

	Definitely agree	Somewhat agree	Somewhat disagree	Definitely disagree
a. People in this neighborhood help each other out	☐	☐	☐	☐
b. We watch out for each other's children in this neighborhood	☐	☐	☐	☐
c. This child is safe in our neighborhood	☐	☐	☐	☐
d. When we encounter difficulties, we know where to go for help in our community	☐	☐	☐	☐
e. This child is safe at school	☐	☐	☐	☐

I15 Other than you or other adults in your home, is there at least one other adult in this child's school, neighborhood, or community who knows this child well and who they can rely on for advice or guidance?

☐ Yes
☐ No

INFORMATIONAL COPY

I16 **The next questions are about events that may have happened during this child's life. These things can happen in any family, but some people may feel uncomfortable with these questions. You may skip any questions you do not want to answer.**

To the best of your knowledge, has this child EVER experienced any of the following?

	Yes	No
**a.** Parent or guardian divorced or separated	☐	☐
**b.** Parent or guardian died	☐	☐
**c.** Parent or guardian served time in jail or prison	☐	☐
**d.** Saw or heard parents or adults slap, hit, kick, punch one another in the home	☐	☐
**e.** Was a victim of violence or witnessed violence in their neighborhood	☐	☐
**f.** Lived with anyone who was mentally ill, suicidal, or severely depressed	☐	☐
**g.** Lived with anyone who had a problem with alcohol or drugs	☐	☐
**h.** Treated or judged unfairly because of their race or ethnic group	☐	☐
**j.** Treated or judged unfairly because of a health condition or disability	☐	☐

I17 **When your family faces problems, how often are you likely to do each of the following?**

	All of the time	Most of the time	Some of the time	None of the time
**a.** Talk together about what to do	☐	☐	☐	☐
**b.** Work together to solve our problems	☐	☐	☐	☐
**c.** Know we have strengths to draw on	☐	☐	☐	☐
**d.** Stay hopeful even in difficult times	☐	☐	☐	☐

I18 **DURING THE PAST 12 MONTHS, has this child had any health care visits by video or phone?**

☐ Yes ☐ No

J. Child's Caregivers

About You

J1 **How are you related to this child?**

☐ Biological or Adoptive Parent
☐ Step-parent
☐ Grandparent
☐ Foster Parent
☐ Other: Relative
☐ Other: Non-Relative

J2 **What is your sex?**

☐ Male
☐ Female

J3 **What is your age?**

☐☐ Age in years

J4 **Where were you born?**

☐ In the United States
☐ Outside of the United States

J5 **What is the highest grade or level of school you have completed?**
Mark (X) ONE box.

☐ 8th grade or less
☐ 9th-12th grade; No diploma
☐ High School Graduate or GED Completed
☐ Completed a vocational, trade, or business school program
☐ Some College Credit, but no Degree
☐ Associate Degree (AA, AS)
☐ Bachelor's Degree (BA, BS, AB)
☐ Master's Degree (MA, MS, MSW, MBA)
☐ Doctorate (PhD, EdD) or Professional Degree (MD, DDS, DVM, JD)

INFORMATIONAL COPY

J6 **What is your marital status?**

- ☐ Married
- ☐ Not married, but living with a partner
- ☐ Never Married
- ☐ Divorced
- ☐ Separated
- ☐ Widowed

J7 **In general, how is your physical health?**

- ☐ Excellent
- ☐ Very good
- ☐ Good
- ☐ Fair
- ☐ Poor

J8 **In general, how is your mental or emotional health?**

- ☐ Excellent
- ☐ Very good
- ☐ Good
- ☐ Fair
- ☐ Poor

J9 **Which of the following best describes your current employment status?**
Mark (X) ONE box.

- ☐ Employed full-time
- ☐ Employed part-time
- ☐ Working WITHOUT pay
- ☐ Not employed but looking for work
- ☐ Not employed and not looking for work
- ☐ Retired

J10 **Have you ever served on active duty in the U.S. Armed Forces, Reserves, or the National Guard?**
Mark (X) ONE box.

- ☐ Never served in the military ➔ ***SKIP to question*** **J12**
- ☐ Only on active duty for training in the Reserves or National Guard ➔ ***SKIP to question*** **J12**
- ☐ Now on active duty
- ☐ On active duty in the past, but not now

J11 **Were you deployed at any time during this child's life?**

- ☐ Yes
- ☐ No

J12 **Does this child have another parent or adult caregiver who lives in this household?**

- ☐ Yes ➔ ***Complete questions*** **J13** - **J23** ***for this other parent or adult caregiver***
- ☐ No ➔ ***SKIP to question*** **K1** ***on page 18***

Other Parent or Caregiver in the Household

J13 **How is this other caregiver related to this child?**

- ☐ Biological or Adoptive Parent
- ☐ Step-parent
- ☐ Grandparent
- ☐ Foster Parent
- ☐ Other: Relative
- ☐ Other: Non-Relative

J14 **What is this caregiver's sex?**

- ☐ Male
- ☐ Female

J15 **What is this caregiver's age?**

☐☐ Age in years

J16 **Where was this caregiver born?**

- ☐ In the United States
- ☐ Outside of the United States

INFORMATIONAL COPY

J17 **What is the highest grade or level of school this caregiver has completed?**
Mark (X) ONE box.

- ☐ 8th grade or less
- ☐ 9th-12th grade; No diploma
- ☐ High School Graduate or GED Completed
- ☐ Completed a vocational, trade, or business school program
- ☐ Some College Credit, but no Degree
- ☐ Associate Degree (AA, AS)
- ☐ Bachelor's Degree (BA, BS, AB)
- ☐ Master's Degree (MA, MS, MSW, MBA)
- ☐ Doctorate (PhD, EdD) or Professional Degree (MD, DDS, DVM, JD)

J18 **What is this caregiver's marital status?**

- ☐ Married
- ☐ Not married, but living with a partner
- ☐ Never Married
- ☐ Divorced
- ☐ Separated
- ☐ Widowed

J19 **In general, how is this caregiver's physical health?**

- ☐ Excellent
- ☐ Very good
- ☐ Good
- ☐ Fair
- ☐ Poor

J20 **In general, how is this caregiver's mental or emotional health?**

- ☐ Excellent
- ☐ Very good
- ☐ Good
- ☐ Fair
- ☐ Poor

J21 **Which of the following best describes this caregiver's current employment status?**
Mark (X) ONE box.

- ☐ Employed full-time
- ☐ Employed part-time
- ☐ Working WITHOUT pay
- ☐ Not employed but looking for work
- ☐ Not employed and not looking for work
- ☐ Retired

J22 **Has this caregiver ever served on active duty in the U.S. Armed Forces, Reserves, or the National Guard?**
Mark (X) ONE box.

- ☐ Never served in the military ➔ ***SKIP to question* K1**
- ☐ Only on active duty for training in the Reserves or National Guard ➔ ***SKIP to question* K1**
- ☐ Now on active duty
- ☐ On active duty in the past, but not now

J23 **Was this caregiver deployed at any time during this child's life?**

- ☐ Yes
- ☐ No

K. Household Information

K1 **How many people are living or staying at this address?**
Include everyone who usually lives or stays at this address. Do NOT include anyone who is living somewhere else for more than two months, such as a college student living away or someone in the Armed Forces on deployment.

☐☐ Number of people

K2 **How many of these people in your household are family members?** *Family is defined as anyone related to this child by blood, marriage, adoption, or through foster care.*

☐☐ Number of people

INFORMATIONAL COPY

K3 **Income in 2022**

Mark (X) the "Yes" box for EACH type of income this child's family received, and give your best estimate of the TOTAL AMOUNT IN THE LAST CALENDAR YEAR. Mark (X) the "No" box to show types of income NOT received.

a. Wages, salary, commissions, bonuses, or tips for all jobs.

☐ Yes ➔ $ ☐,☐☐☐,☐☐☐.00

☐ No

TOTAL AMOUNT in the last calendar year

b. Self-employment income from own nonfarm businesses or farm business, including proprietorships and partnerships.

☐ Yes ➔ $ ☐,☐☐☐,☐☐☐.00 ☐ Loss

☐ No

TOTAL AMOUNT in the last calendar year

c. Interest, dividends, net rental income, royalty income, or income from estates and trusts.

☐ Yes ➔ $ ☐,☐☐☐,☐☐☐.00 ☐ Loss

☐ No

TOTAL AMOUNT in the last calendar year

d. Social Security or Railroad Retirement; retirement, survivor, or disability pensions.

☐ Yes ➔ $ ☐,☐☐☐,☐☐☐.00

☐ No

TOTAL AMOUNT in the last calendar year

e. Supplemental Security Income (SSI); any public assistance or welfare payments from the state or local welfare office.

☐ Yes ➔ $ ☐,☐☐☐,☐☐☐.00

☐ No

TOTAL AMOUNT in the last calendar year

f. Any other sources of income received regularly such as Veterans' (VA) payments, unemployment compensation, child support, or alimony.

☐ Yes ➔ $ ☐,☐☐☐,☐☐☐.00

☐ No

TOTAL AMOUNT in the last calendar year

K4 **The following question is about your 2022 income. Think about your total combined family income IN THE LAST CALENDAR YEAR for all members of the family. What is that amount before taxes?** *Include money from jobs, child support, social security, retirement income, unemployment payments, public assistance, and so forth. Also, include income from interest, dividends, net income from businesses, farm or rent, and any other money income received.*

$ ☐,☐☐☐,☐☐☐.00 ☐ Loss

TOTAL AMOUNT in the last calendar year

INFORMATIONAL COPY

Mailing Instructions

Thank you for your participation.

On behalf of the U.S. Department of Health and Human Services, we would like to thank you for the time and effort you have spent sharing information about this child and your family.

Your answers are important to us and will help researchers, policymakers, and family advocates to better understand the health and health care needs of children in our diverse population.

Place the completed questionnaire in the postage-paid return envelope. If the envelope has been misplaced, mail the questionnaire to:

U.S. Census Bureau
ATTN: DCB 60-A
1201 E. 10th Street
Jeffersonville, IN 47132-0001

INFORMATIONAL COPY

We estimate that completing the National Survey of Children's Health will take 35 minutes on average. Send comments regarding this burden estimate or any other aspect of this collection of information, including suggestions for reducing this burden, to U.S. Department of Commerce, Paperwork Project 0607-0990, U.S. Census Bureau, 4600 Silver Hill Road, Room 8H590, Washington, DC 20233. You may e-mail comments to DEMO.Paperwork@census.gov; use "Paperwork Project 0607-0990" as the subject. This collection has been approved by the Office of Management and Budget (OMB). The eight-digit OMB approval number that appears at the upper left of the form confirms this approval. If this number were not displayed, we could not conduct this survey.
